# Deep surgical site infection after posterior instrumented fusion for rheumatoid upper cervical subluxation treated with antibiotic-loaded bone cement: Three case reports

**DOI:** 10.1097/MD.0000000000020892

**Published:** 2020-06-26

**Authors:** Satoshi Ogihara, Shuhei Murase, Fumihiko Oguchi, Kazuo Saita

**Affiliations:** a Department of Orthopaedic Surgery, Spine Center, Sagamihara National Hospital, Minami-ku, Sagamihara City, Kanagawa, Japan; b Department of Orthopaedic Surgery, Saitama Medical Center, Saitama Medical University, Kawagoe, Saitama, Japan.

**Keywords:** bone cement, instrumented fusion, rheumatoid arthritis, surgical site infection, upper cervical spine

## Abstract

**Introduction:**

Patients with rheumatoid arthritis (RA) tend to be immunosuppressed due to RA itself and the therapeutic drugs administered. The management of surgical site infection (SSI) following upper cervical spinal instrumented fusion in RA patients is challenging; however, literature on the treatment for such conditions is scarce. We report 3 consecutive patients with RA, who developed deep SSI following upper cervical posterior fusion and were treated using antibiotic-loaded bone cement (ALBC).

**Patient Concerns:**

All 3 patients reported in the current study experienced compression myelopathy with upper cervical spinal deformity and received prednisolone and methotrexate for controlling RA preoperatively. The patient in Case 1 underwent C1–2 posterior fusion and developed deep SSI due to methicillin-sensitive *Staphylococcus aureus* at 3 months postoperatively; the patient in Case 2 underwent occipito-C2 posterior fusion and developed deep SSI due to methicillin-sensitive *Staphylococcus aureus* at 2 weeks postoperatively; and the patient in Case 3 underwent occipito-C2 posterior instrumented fusion and laminoplasty at C3–7, and developed deep SSI due to methicillin-resistant coagulase negative staphylococci at 3 weeks postoperatively.

**Diagnosis:**

All patients developed deep staphylococcal SSI in the postoperative period.

**Interventions:**

All 3 patients were treated using ALBC placed on and around the instrumentation to cover them and occupy the dead space after radical open debridement.

**Outcomes:**

The deep infection was resolved uneventfully after the single surgical intervention retaining spinal instrumentation. Good clinical outcomes of the initial surgery were maintained until the final follow-up without recurrence of SSI in all 3 cases.

**Conclusion:**

ALBC embedding spinal instrumentation procedure can be a viable treatment for curing SSI in complex cases, such as patients with RA who undergo high cervical fusion surgeries without implant removal.

## 1 Introduction

Atlantoaxial subluxation (AAS) and vertical subluxation (VS) are known upper cervical spine manifestations of rheumatoid arthritis (RA) that cause cervical instability and compression myelopathy. With recent advances in spinal instrumentation surgery, posterior fusion for deformities of the upper cervical spine has been performed more frequently, reportedly with good clinical outcomes.^[1,2]^ However, patients with RA are susceptible to infections because of the immunosuppressive state from the RA disease process and the therapeutic drugs for RA. They are reported to be at high risk of surgical site infection (SSI) following spinal fusion surgeries.^[3,4]^ Additionally, only limited literature is available on the treatment for SSI after instrumented fusion for upper cervical spine deformity in patients with RA.^[5–7]^

Here, we report 3 consecutive patients with RA who developed deep staphylococcal SSI following posterior instrumented fusion for upper cervical spine deformity. They were successfully treated with a single surgical procedure using antibiotic-loaded bone cement (ALBC) while preserving spinal instrumentation. We also review the relevant literature.

## 2 Case report

This study was approved by the institutional review board of Sagamihara National Hospital, Kanagawa, Japan. Written informed consent was obtained from all patients for publication of this case report and accompanying images.

### 2.1 Case 1

**Figure 1:**
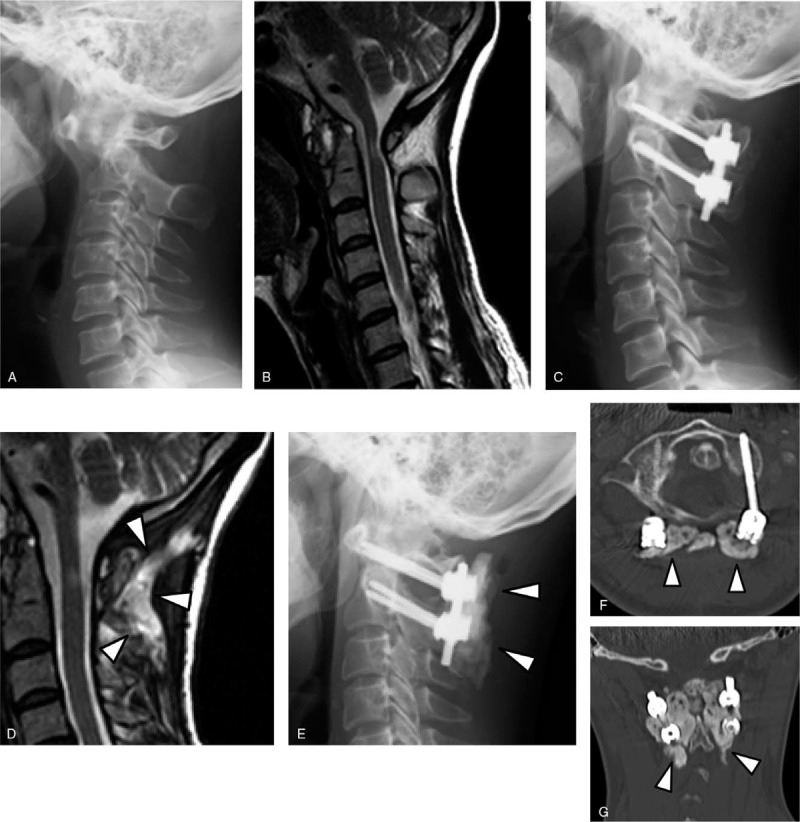
Case 1: (A) Preoperative lateral view radiograph. (B) Preoperative mid-sagittal T2-weighted MRI. (C) Postoperative lateral view radiograph. (D) Mid-sagittal T2-weighted MRI taken after the occurrence of deep SSI, revealing abscess formation in the subfascial space (*arrowhead*s). (E) Lateral view radiograph recorded following the procedure for the treatment of deep SSI. ALBC covering metal implants is shown (*arrowhead*s). (F) Axial CT image and (G) coronal CT image following the procedure for the treatment of deep SSI. The images show that ALBC embedded metal implants occupy the dead space in the operated wound (*arrowhead*s). ALBC = antibiotic-loaded bone cement, CT = computed tomography, MRI = magnetic resonance imaging, SSI = surgical site infection.

A 44-year-old woman with RA (onset age: 19 years) was treated with prednisolone (PSL) 2 mg/day and methotrexate (MTX) 3 mg/week preoperatively for RA management. She reported persistent pain in her posterior neck and head, and numbness and clumsiness in both hands. Radiographs showed reducible AAS (Fig. 1A). Magnetic resonance imaging (MRI) revealed a high signal intensity change and atrophy of the spinal cord at the C1–2 level (Fig. 1B). We performed C1–2 posterior fusion using bilateral C1 lateral mass screws, C2 pedicle screws, and iliac bone graft for treatment of compression myelopathy due to AAS (Fig. 1C). Immediately after the surgery, her preoperative clinical symptoms improved.

At 2 months postoperative, spontaneous dehiscence of the surgical wound was observed, and purulent exudate was discharged from the opened portion of the wound. MRI revealed abscess formation in the subfascial space around the spinal instrumentation (Fig. 1D). Preoperative microbiological culture was taken from the discharged fluid, and gram-positive cocci were identified by Gram staining.

Surgical treatment using ALBC was performed (Figs. 1E, F, G). Specimens for microbiological culture study were collected intraoperatively from the subfascial infected tissues. The Gram staining procedure identified gram-positive cocci. Microbiological cultures revealed the same strain of methicillin-sensitive *Staphylococcus aureus* from the preoperative and intraoperative specimens. After the single surgical intervention using ALBC, the deep infection was resolved uneventfully. C-reactive protein (CRP) and erythrocyte sedimentation rate (ESR) levels decreased to the preoperative levels 2 weeks after the surgical treatment for deep SSI. She maintained a good clinical outcome of the initial surgery until the final follow-up at 93 months postoperatively, without recurrence of SSI.

### 2.2 Case 2

**Figure 2:**
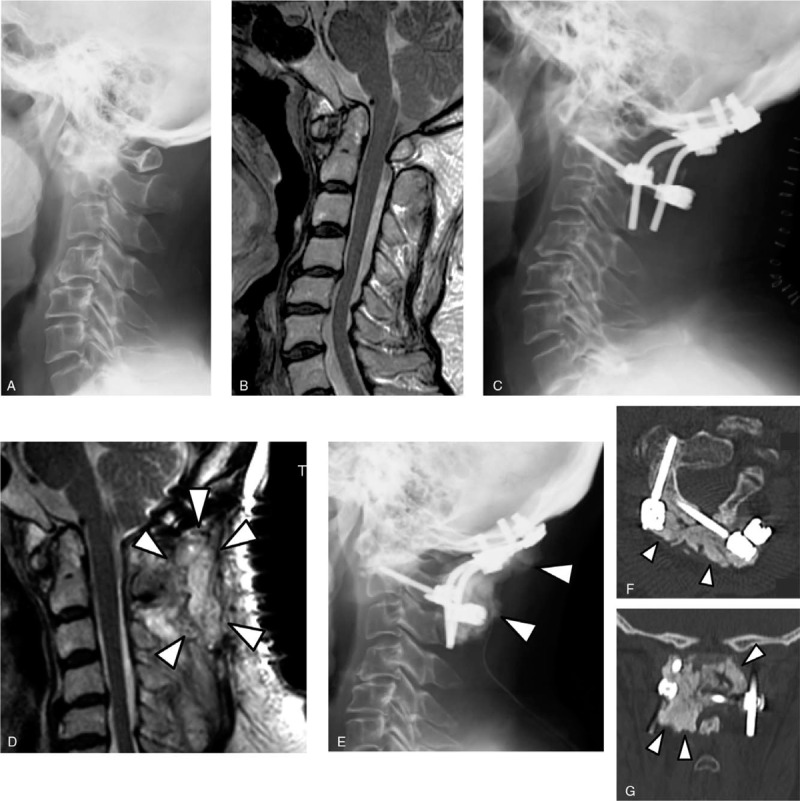
Case 2: (A) Preoperative lateral view radiograph. (B) Preoperative mid-sagittal T2-weighted MRI. (C) Postoperative lateral view radiograph. (D) Mid-sagittal T2-weighted MRI taken after the occurrence of deep SSI, revealing abscess formation in the subfascial space around the spinal instrumentation (*arrowhead*s). (E) Lateral view radiograph recorded following the procedure for the treatment of deep SSI. ALBC covering metal implants is shown (*arrowhead*s). (F) Axial CT image and (G) coronal CT image following the procedure for the treatment of deep SSI. The images show that ALBC embedded metal implants occupy the dead space in the operated wound (*arrowhead*s). ALBC = antibiotic-loaded bone cement, CT = computed tomography, MRI = magnetic resonance imaging, SSI = surgical site infection.

A 61-year-old woman with RA (onset age: 52 years) was treated with PSL 9 mg/day and MTX 8 mg/week preoperatively for RA management. She reported severe persistent pain in her left posterior neck and head and paresthesia in both hands. Radiographs showed atlantoaxial VS (Fig. 2A). MRI revealed an atlantoaxial VS and compression of the spinal cord at the C1–2 level (Fig. 2B). Occipito-C2 posterior fusion was performed using an occipital plate, C2 pedicle screw on the right side, C2 laminar screw on the left side, and an iliac bone graft (Fig. 2C). Immediately after surgery, her preoperative clinical symptoms improved.

At 2 weeks postoperative, spontaneous dehiscence of the surgical wound and purulent exudate from the opened portion of the wound were observed, and she developed a fever greater than 38°C. MRI revealed abscess formation in the deep subfascial space around the spinal instrumentation (Fig. 2D). Microbiological culture was taken from the discharged fluid; the Gram staining procedure revealed gram-positive cocci.

Surgical treatment using ALBC was performed (Figs. 2E, F, G). Specimens for microbiological culture were taken intraoperatively from the subfascial infected tissues. The Gram staining procedure identified gram-positive cocci, while the microbiological culture revealed the same strain of methicillin-sensitive *Staphylococcus aureus* from the preoperative and intraoperative specimens. The deep infection resolved uneventfully through this single surgical intervention using ALBC, and CRP and ESR levels decreased to the preoperative level at 2 weeks after the surgical treatment for deep SSI. She maintained a good clinical outcome of the initial surgery up to the final follow-up at 91 months postoperative without recurrence of SSI.

### 2.3 Case 3

**Figure 3:**
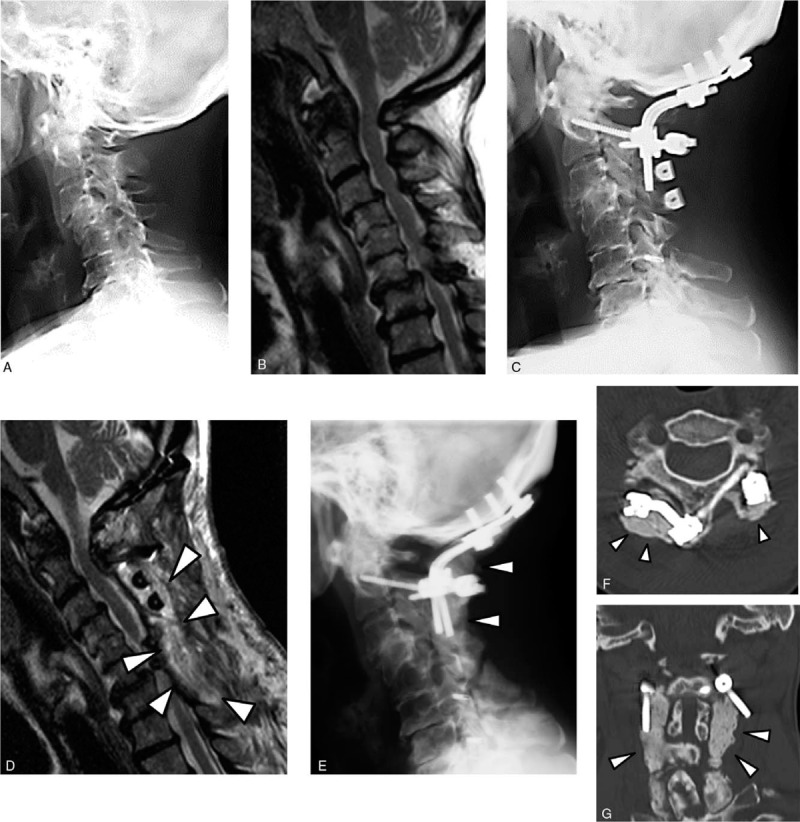
Case 3: (A) Preoperative lateral view radiograph. (B) Preoperative mid-sagittal T2-weighted MRI. (C) Postoperative lateral view radiograph. (D) Mid-sagittal T2-weighted MRI taken after the occurrence of deep SSI, revealing abscess formation in the subfascial space (*arrowhead*s). (E) Lateral view radiograph recorded following the procedure for the treatment of deep SSI. ALBC covering metal implants is shown (*arrowhead*s). (F) Axial CT image and (G) coronal CT image following the procedure for the treatment of deep SSI. The images show that ALBC embedded metal implants occupy the dead space in the operated wound (*arrowhead*s). ALBC = antibiotic-loaded bone cement, CT = computed tomography, MRI = magnetic resonance imaging, SSI = surgical site infection.

A 72-year-old woman with RA (onset age: 53 years) was treated with MTX 6 mg/wk and PSL 7 mg/d preoperatively for RA management. She reported numbness and clumsiness in both hands and a gait disturbance. Her walking ability had gradually declined, and she required a wheelchair 3 months after symptom onset. Radiographs showed irreducible AAS and deformation at the mid and lower cervical spine, including spondylolisthesis at C3–4 and C6–7 levels (Fig. 3A). MRI revealed compression of the spinal cord at C1–2, C3–4, and C6–7 levels (Fig. 3B). Occipito-C2 posterior fusion (using an occipital plate, C2 laminar screw on the right side, C2 pedicle screw on the left side, and iliac bone graft), double door laminoplasty using hydroxyapatite spacers at the C3–4 levels, and open door laminoplasty at the C5–7 levels were performed (Fig. 3C).

At 3 weeks postoperative, spontaneous dehiscence of the surgical wound and purulent exudate from the opened portion of the wound were observed, and she developed persistent neck pain. MRI revealed abscess formation around the deep subfascial space (Fig. 3D). A microbiological culture was taken from the discharged fluid. The Gram staining procedure revealed gram-positive cocci.

Surgical treatment using ALBC was performed (Figs. 3E, F, G). Specimens for microbiological culture were taken intraoperatively from the subfascial infected tissues. The Gram staining procedure identified gram-positive cocci. The microbiological culture revealed the same strain of methicillin-resistant coagulase negative staphylococci from the preoperative and intraoperative specimens. After the single surgical procedure using ALBC, the deep infection was resolved uneventfully. Her CRP and ESR levels decreased to the preoperative level at 1 month after surgical treatment for deep SSI. Two weeks after the second operation, the patient could walk without aid and she maintained a good clinical outcome of the index surgery without recurrence of SSI up to the final follow-up at postoperative 11 months, when she died from hemorrhagic cerebral infarction.

### 2.4 Surgical technique

Within 2 days from the time of the diagnosis of deep SSI, the patients were transferred to the operating room for surgical treatment. Meticulous open debridement of infected tissues, removal of the grafted bone, and large-volume irrigation using normal saline were performed. In the 3 current cases, the preoperative Gram stain procedures identified gram-positive cocci. Thus, we selected vancomycin (VCM) as the antibiotic drug mixed with the bone cement. To make ALBC, the bone cement was mixed with VCM powder (3.0 g of VCM per 40 g of bone cement). The exposed surface of the instrumentation was embedded, and dead space in the surgical field was filled with ALBC. A closed suction drain was placed subfascially, and the wound was primarily closed. Intravenous administration of antibiotics was started with VCM. In Case 1 and Case 2, the intravenous antibiotic was changed to cefazolin based on the results of the microbiological culture. The intravenous administration of antibiotic was continued until 8 weeks postoperative in all 3 cases. The drainage was removed within 7 days postoperative. We did not restrict patients’ activity after the procedure. All patients were discharged after finishing intravenous administration of antibiotics. ALBC was placed *in situ* in all patients during the follow-up period.

## 3 Discussion

Patients with RA are known to be susceptible to infections because of the immunosuppressive state from the RA disease process and the medications used in the management for RA, such as steroids^[8]^ and disease-modifying antirheumatic drugs (eg, MTX^[9]^). Immunosuppression in patients with RA is considered to have a negative influence on the management and cure of infections. Patients with RA are reported to be at higher risk of SSI after spinal surgeries and prosthetic joint replacement surgeries compared to the general population.^[3,4,10]^ Furthermore, Koyama et al^[11]^ reported that the use of instrumentation was an independent risk factor for SSI following spinal surgery in patients with RA.

When deep SSI occurs in patients with RA who undergo a high cervical spinal fusion procedure, the management of SSI is challenging. A biofilm may form around the surface of the implants in case of deep wound infection,^[12]^ and the minimal bactericidal concentration of antibiotics after biofilm formation is reported to be 100- to 1000-fold higher^[12]^; thus, implant removal is considered to be advantageous to cure deep SSI. However, implant removal may lead to devastating spinal reconstruction failure of the upper cervical spine, which can lead to poor clinical outcome, including exacerbation of instability at the upper cervical spine and critical deterioration of high-cervical compression myelopathy. Therefore, in patients with RA who develop deep SSI after high-cervical posterior fusion, curing SSI while retaining spinal instrumentation has great clinical significance.

However, there have been only a few reports describing patients suffering from RA who developed SSI after instrumentation fusion for upper cervical spine deformities.^[5–7]^ There is also a paucity of previous literature with detailed information on the practical treatment for SSI following upper cervical instrumented fusion and its clinical outcome. Nagaria et al^[5]^ reported that SSI occurred in 8.1% (3 of 37) of patients with RA who underwent C1-C2 transarticular screw fixation for AAS. Hirano et al^[6]^ described that SSI occurred in 14.3% (8 of 56) of patients who underwent occipitothoracic fusion, and 37.5% (3 of 8) of the infected patients were treated by implant removal. Zygmunt et al^[7]^ described that SSI occurred in 20.3% (16 of 79) of patients who underwent occipitocervical fusion, while minor infections occurred in 11 of 16, and 100% (5 of 5) of patients who developed deep SSI were treated by implant removal.

To avoid implant removal that could cause poor clinical outcome, several surgical methods have been reported. Repetitive surgical debridement, combined with the use of systemic antibiotics, is the most frequently recommended treatment strategy.^[13,14]^ Mok et al reported that an extensive treatment period and repeated surgical procedures were needed for resolution of deep SSI after lumbar instrumented fusion, and 25% of patients with SSI in the study required implant removal.^[14]^ However, repeated debridement with general anesthesia may be particularly disadvantageous for patients with RA. They often have comorbidities, such as interstitial lung diseases^[15]^ and cardiovascular disorders,^[16]^ and their ability to endure multiple surgeries is relatively low. Thus, less invasive surgical treatments are preferred, and avoiding multiple surgeries as much as possible is desirable. Additionally, treatment methods of closed-suction irrigation system^[17]^ and vacuum-assisted closure^[18]^ are reported for spinal SSI. However, these methods are frequently associated with prolonged restriction of activities for patients and necessitate an extensive treatment period and additional manpower in many cases. Two-stage revision arthroplasty with interval placement of an ALBC spacer is widely accepted as a standard method for the treatment of chronic infection after joint replacement surgery.^[19]^ However, there are only 3 previous reports describing the ALBC embedding instrumentation procedure for treatment of spinal SSI with a good outcome while retaining metal implants.^[20–22]^

Gram-positive organisms (including *Staphylococcus*, *Streptococcus*, etc) are known to be predominant pathogens in spinal SSI.^[23–25]^ The Gram staining procedure provides results faster than culture and is especially important when timely management of the infection would make an important difference in the patient's treatment. When Gram staining reveals gram-positive organisms as the causative pathogen and the results of culture inspection has not arrived yet, we consider VCM to be an effective drug for mixing in bone cement to make the ALBC, because gram-positive bacteria are widely sensitive to VCM, including multidrug-resistant organisms, such as methicillin resistant *Staphylococcus aureus* and methicillin-resistant coagulase negative staphylococci. Additionally, VCM is relatively thermostable and has been selected for loading in bone cement in many past reports regarding treatment for SSI.^[20–22,26]^ Aminoglycosides are also frequently used for impregnation of bone cement, and the aminoglycoside-VCM combination in bone cement is reported to have a wide antimicrobial spectrum and act synergistically to increase the elution rates of both drugs.^[26]^ In this case series, we used only VCM to make the ALBC, but we consider that using aminoglycoside combined with VCM for ALBC may provide an additive effect for the control of infection.

The addition of the ALBC embedding instrumentation procedure to conventional intravenous antibiotic administration and infected tissue debridement has several advantages in the treatment of SSI in spinal surgeries. First, ALBC elutes antibiotics over a period of time and maintains a local concentration of antibiotic drug targeting the causative organisms with minimal systemic toxicity.^[27]^ Second, the placement of ALBC around the implants reduces the dead space in the operated wound. Dead space in the surgical wound is associated with the formation of hematoma or seroma, which increases the risk of bacterial growth.^[25]^ Third, this method is easy to perform and cost-effective. The ALBC embedding instrumentation procedure does not necessitate prolonged bed rest and additional operations for bone cement removal. In fact, SSI was resolved by a single surgical intervention without restricting patient activity after the procedure in the current patients. Furthermore, this method may provide the reinforcement for spinal column stability of the affected segments where the grafted bone was removed during the debridement procedure.^[28]^ In our case series, instrumentation embedded with ALBC at the affected segment has maintained stability during the follow-up period (especially in Case 1 and Case 2: over 7 years) without any adverse events.

This report has some limitations, including the nature of the retrospective study, which was performed in a single institution, and the small number of patients. Despite these limitations, we consider that this report provides important information concerning salvage treatment for patients with RA who undergo high cervical instrumented fusion complicated by SSI. Further research to validate efficacy of the ALBC embedding spinal instrumentation method is needed.

In conclusion, the ALBC embedding spinal instrumentation technique provides reduction of dead space and delivery of targeted drug simultaneously. Therefore, it can be considered a viable method for preserving metal implants in complex cases, such as patients with RA who undergo high cervical instrumented fusion procedures complicated by SSI.

## Author contributions

**Conceptualization:** Satoshi Ogihara.

**Data curation:** Shuhei Murase, Satoshi Ogihara.

**Investigation:** Satoshi Ogihara, Fumihiko Oguchi.

**Supervision:** Kazuo Saita.

**Writing – original draft:** Satoshi Ogihara.

**Writing – review & editing:** Satoshi Ogihara.
